# Multi-center survey of House officers’ choice of Medical specialties in Nigeria: preferences and determining factors

**DOI:** 10.11604/pamj.2015.20.338.4113

**Published:** 2015-04-08

**Authors:** Kelechi Emmanuel Okonta, Idorenyin Cletus Akpayak, Ezekiel Olatunde Amusan, Eyo Effiong Ekpe, Yahaya Baba Adamu, Emmanuel Ossai Ocheli

**Affiliations:** 1 Department of Surgery, University of Port Harcourt Teaching Hospital, Rivers State, Nigeria; 2 Department of Surgery, Jos University Teaching Hospital, Plateau State, Nigeria; 3 Department of Surgery, University of Uyo Teaching Hospital, Akwa Ibom State, Nigeria; 4 Department of Surgery, National Hospital Abuja, Nigeria

**Keywords:** House officer, Medical specialty, determining factors, tertiary hospital

## Abstract

The objective of the study was to determine preferences and factors influencing the choice of medical specialties by House officers. Questionnaires were distributed to House-officers in 4 tertiary hospitals namely: the National hospital, Abuja, the University of Port-Harcourt, the Jos University, and the University of Uyo Teaching Hospitals. The data were simultaneously collected and analyzed using SPSS 20.0 version. Of the 150 questionnaires distributed, 129(86%) were duly filled. The mean age was 22.4 years (range 21-40 years), 79(61.2%) of the respondent were male. Fifty-nine(45.7%) chose training within the country while 32(24.8%) preferred outside as 107(86%) chose training in Teaching Hospitals. Teachers, Resident doctors and parents influenced choices in 34(26.3%), 17(13.1%) and 16(12.4%) respectively. Thirty-four(26.3%), 28 (21.7%), 13(10.1%) and 15(11.6%) preferred obstetrics, surgery, internal medicine and paediatrics respectively. Seventy (46.7%) chose specialties for personal likeness and 17(11.3%) for role models in that specialty. House officers preferred to pursue medical specialty in teaching hospitals within the country and they are motivated by personal fulfillment, independence of practice and role models while more prefer to specialize in more Obstetrics/ Gyaenocology and surgery.

## Introduction

Immediately after graduation, newly graduated Medical Doctors are groomed for the onerous task of specializing in different medical fields in the country's tertiary health institutions in other to satisfy the country's apt and yearning health care needs. This will subsequently ensure that the patients receive specialist care; help in sharpening the Doctors acumen for focused clinical research in different specialized areas and, subsequently, lead to the formulation and implementation of commensurate health care programs [1, 2]. Also, the factors responsible for Doctors in making the necessary choices of medical specialties are important as this will lead to the development of ways that will positively influence the practice of medicine [1]. There are various studies conducted within and outside the country to highlight the factors influencing the choice of medical specialties [1–18] but, the main limitation is that, most of these studies were single centre based studies [1–9]. The process of choosing medical specialties and the subsequent posting of………. graduating Doctors to such areas requires utmost care, as improper arrangements, may not satisfy the local communities’ specialized health care needs leading to shortage of required physicians in some specialties in such countries [16]. Some specialties like otorhinolaryngology, ophthalmology and community medicine were less chosen when compared to other specialties like surgery, pediatrics, internal medicine and obstetrics/ gynaecology [1, 2, 6–8]. The process of choosing a medical specialty, in the country, requires a post-graduate training in the country; a mandatory six year program of basic medical training which comprises of one year of basic sciences, two years of basic medical sciences and three years of clinical sciences. Thereafter, a 1-year compulsory internship which involves rotation in the four major departments of Internal medicine, surgery, pediatrics and obstetrics and gynecology; and National Youth Service Corps program for 1 year. The residency training involves a pass in primaries (basic medical and clinical sciences), Part I and Part II examinations organized by National Post-graduate Medical College of Nigeria and West African Post graduate Medical College [19]. There are 31 medical/dental schools in Nigeria. Twenty seven are fully accredited while 4 are partially accredited and they have student quota ranging from 50 to 150 [20]. There are 91 hospitals accredited for internship training [20]. The establishment of many specialist centers in Nigeria, makes residency training easily accessible. To determine preferences and factors influencing the choice of medical specialties by House-offers.

## Methods

The study was a multi-center cross-sectional survey of House officers in four tertiary institutions in Nigeria namely: the University of Port Harcourt Teaching Hospital, Port-Harcourt; the University of Uyo Teaching Hospital, Uyo; the National Hospital, Abuja and Jos University Teaching Hospital, Jos. The study was simultaneously conducted. A structured questionnaire was administered to collect data on the participants’ age, sex, whether they will specialize, the time the decision of specialization was made, the choice of medical specialty, the reasons for their choices, the preferred location and institution for their training and the individuals who influenced their choice. The data were analyzed using Statistical package for social sciences (SPSS) software version 20.0 (Inc. Chicago, IL).

## Results

Out of 150 structured questionnaires distributed to the centers, 129(86%) were properly filled and returned while 21(14%) Participants did not respond to most questions and were therefore excluded from the study. Seventy nine participants (61.2%) were male and 50 (38.8%) were female with the age range 21 to 40 years. The mean age was 22.4 years (range 21-25 years) (Table 1]. Forty seven (36.4%) participants made their choices in the course of their clinical posting, 47(36.4%) during housemanship, 21(16.3% during the pre-clinical posting while 14(10.9%) participants responded that their choice would be made after the housemanship (Figure 1). Thus, 78(60.5%) made definite decision to specialize while 51 (39.5%) did not. Fifty nine (45.7%) chose doing their specialization within the country while 32 (24.8%) preferred outside the country. Thirty six (27.9%) did not decide where they will do their training and 2 (1.6%) did not specify their training location.

**Table 1:** Age and sex distribution of participants

Age Group	Male (%)	Female (%)
21 – 25	36 (27.9)	36 (27.9)
26 – 30	32 (24.8)	14 (10.8)
31 – 35	4 (3.1)	- (0)
36 – 40	2 (1.5)	- (0)
Not specified	5 (3.8)	-
**Total**	**79(61.2)**	**50 (38.8)**

One hundred and eleven (86%) chose to undergo the training in the Teaching Hospitals; 10(7.8%) and 1(0.8%) chose Federal medical centers and private hospitals respectively with 7(5.4%) did not know which category of hospital their training will be done (Table 2). The Teachers, the Resident doctors and the Parents influenced 34(26.3%), 17 (13.1%) and 16 (12.4%) respectively in choosing medical specialties (Table 3). Thirty four (26.3%) preferred obstetrics and gynecology, while 28 (21.7%), 13 (10.1%) and 15 (11.6%) preferred surgery, internal medicine and pediatrics respectively. Other preferences were community medicine in 6(4.6%) participants, 7(5.4%) in family medicine while ophthalmology and pathology had 4 (3.1%) participants each with 7(5.4%) remaining undecided about their choice of medical specialty. Seventy (46.7%) participant chose a specific specialty because of personal interest, while 17(11.3%) were influenced by the presence of role models in the specialties, 14(9.3%) by expected financial reward and 16(10.7%) because of independence of practice and 6(4.0%) house officers were influenced by their families (Table 4).

**Table 2:** Choice of Institution

Institution	Frequency	%
Teaching Hospital	111	86.0
Federal Medical Centre	10	7.8
General Hospital	-	-
Private Hospital	1	0.8
I don't know	7	5.4
Total	129	100

**Table 3:** Individuals who influenced their choice

Individual	Frequency	%
Colleagues	6	4.6
Teachers	34	26.3
Residents	17	13.1
Parents	16	12.4
Religion	2	1.5
Others	54	41.9
**Total**	**129**	**100**

**Table 4:** Reasons for choosing a particular specialty

Reason	Frequency	Percent
Personal Liking/Flair Speciality	70	46.7
Family Influence	6	4.0
Friends Influence	-	-
Society Perception On Importance Of Speciality	7	4.7
Financial Reward	14	9.3
Independence Of Practice	16	10.7
Training Is Not Strenous	9	6.0
Short Training	6	4.0
Speciality Has Many Role Models	17	11.3
**Total**	**150**	**100**

## Discussion

This is a multi-centre survey consisting of four tertiary hospitals: two from the northern and two from the southern parts of the country. The selection of these centers ensured that there was uniform access and even distribution of the questionnaires to the House officers working at the different hospitals at that moment. The data were also simultaneously collected in other to avoid bias and possible duplication.

The female to male ratio of 1:1.6 is similar to another study done in Nigeria [6]. However, there was slight increase in number of female respondents when compared to the previous studies [7]. This may suggest that the sex imbalance that was peculiar with medical training in the past was changing. The finding showed that most of the respondents made their decisions before they finished their one year internship training. These finding shows that there have not been significant change from previous studies [10–12]. Most of the House officers will opt to do the residency program in a teaching hospital, as was observed in previous study done in the country too [19]. This, of course, has two implications: The first is that there will be more members of staff and students in these teaching hospitals. The other implication is to make a case for proper funding of these hospitals and make them braze up to the challenges occasioned by this expected increase.

Obstetrics/ gynaecology and surgery were the most preferred specialties by the House officers as more than half opted for them, and this can be compared to findings in other parts of the world [1, 3, 15]. In a study done in Jordan amongst medical students it was observed that these two specialties topped in their choices: The females they opted for Obstetrics while the males opted for surgery [3]. A more explicit study done in Malaysia earlier on showed the same gender preference for the specialties [4]. However we did not decipher our selections along gender line to verify this in our own students but sex does have influence on the choice of specialties [4, 7]. Community medicine was chosen less by the interns which collaborate with the findings from previous studies [3, 14, 20]. The impact of choosing community medicine less is more worrisome as it could mean that the emphasis on preventive medicine is not being embraced and that could create a dearth of specialist in this field in future. Thus a critical review by the hospital management and administrators is required to strengthen the fields that are being overlooked by the students [15].

House officers were influenced by their Teachers thus highlighting the impact of the influence of teachers in the choice of medical specialties. It is noted from a study that the innovativeness and dedication on the part of teachers can arouse the interest of students during posting in that particular specialty [2]. The other important influencing factor was the Residents Doctors; who, in actual fact, spend more time with the house officers and Family members [17, 18]. The influence of parents and guardians were not surprising as most families wish to have medical specialists in their family. The influence aspect should be strengthened by proper education. However, the reason for choosing a particular specialty is mainly for personal liking/flair followed by independence of practice. The work done by Eze etal for pre-residency medical graduates in southeastern Nigeria showed that they were influenced by personal interests, career prospects, and personal skills/aptitude in deciding which specialty training to pursue [1]. Other studies also pointed out that the main factors affecting specialty choices were students′ interest in the career [13, 15].

## Conclusion

Most House officers preferred specializing in clinical areas like Obstetrics and Surgery while choosing less of community and internal medicine. The reasons for choosing particular specialty were personal likeness, independence of practice and the presence of role models in the specialty. We therefore advocate that infrastructures be put in place to accommodate these desires and the choices properly guided to avoid dearth of some specialist in future.

## References

[1] Eze BI, Okoye OI, Maduka-okafor FC, Aguwa EN (2011). Factors influencing choice of medical specialty of pre-residency medical graduates in southeastern. Niger J Grad Med Educ..

[2] Ohaeri JU, Akinyinka OO, Azuzu MC (1992). The specialty choice of clinical year students as the Ibadan Medical School. Afr J Med Med Sci..

[3] Khader Y, Al-Zoubi D, Amarin Z, Alkafagei A, Khasawunch M, Burgan S (2008). Factors affecting medical students in formulating their speciality preferences in Jordan. BMC Med Educ..

[4] Zulkifi A, Rogayah J (1997). Career preferences of male and female medical students in Malaysia. Med J Malaysia..

[5] Saigal P, Taakemura Y, Nishiue T, Fetters MD (2007). Factors considered by medical students when formulating their specialty preferences in Japan: findings from a qualitative study. BMC Med educ..

[6] Odusanya OO, Alakija W, Akesode FA (2000). Socio demographic profile and career aspirations of medical students in a new medical school. Niger Postgrad Med J..

[7] Oyebola DD, Adewoye OE (1988). Preference of preclinical medical students for medical specialties and the basic sciences. Afr J Med Med Sci..

[8] Ojo OS, Afolabi ERI (1992). Social issues in medicine: an evaluation of determinants of medical manpower resource. Orient J Med..

[9] Ogbonnaya LU, Agu AP, Nwonwu EU, Ogbonnaya CE (2004). Specialty choice of residents in the University of Nigeria Teaching Hospital. Enugu 1989-19 Orient J Med..

[10] Harris MG, Gavel PH, Young JR (2005). Factors influencing the choice of specialty of Australian medical graduates. Med J Aust..

[11] Davidson JM, Lambert TW, Goldacre MJ (1988). Career pathways and destinations 18 years on among doctors who qualified in the United Kingdom in 1977:Postal Questionnaire survey. BMJ..

[12] Goldacre MJ, Lambert TW (2003). Stability and change in career choice of junior doctors: Postal Questionnaire survey of UK qualifiers of 2003. Med Educ..

[13] Omnia S, Seifi El, Eman Mortada M (2011). Specialty Choices Among Medical Students And Interns In Egyptian Accredited And Non Accredited Faculties Of Medicine. Curr Sc Intern..

[14] Brotherton SE, Rockey PH, Etzel SI (2004). US graduate medical education, 2003-2004. JAMA..

[15] Huda N, Yousuf S (2006). Career preference of final year medical students of Ziauddin Medical University. Educ Health..

[16] Knox KE, Getzin A, Bergum A, McBride P, Riesebalch R, Friedsam D (2008). Short report: factors that affect specialty choice and career plan of Wisonsin's medical students. WMJ..

[17] Corkin D, Arbona C, Coleman N, Ramirez R (2008). Dimensions of career in decision among Puerto Rican college students. J Coll Stud Dev..

[18] Nedjat S, Emami-Razavi H, Rashidian A, Yazdani S, Majdzadeh S (2006). Factors associated with choosing medical school among students of Tehran Faculty of Medicine and their awareness about future of their career: a qualitative-quantitative approach. Steps Dev Med Edu..

[19] The Bulletin of the National Postgraduate Medical College of Nigeria (1999).

[20] Medical and Dental Council of Nigeria (online) http://www.mdcnigeria.org.

